# Association between major depressive disorder and pro-inflammatory cytokines and acute phase proteins among HIV-1 positive patients in Uganda

**DOI:** 10.1186/s12865-017-0239-3

**Published:** 2018-01-03

**Authors:** Kenneth Musinguzi, Andrew Obuku, Noeline Nakasujja, Harriet Birabwa, Juliet Nakku, Jonathan Levin, Eugene Kinyanda

**Affiliations:** 1 MRC/UVRI, Uganda Research Unit on AIDS, P.O. Box 49, Entebbe, Uganda; 2 Department of Psychiatry, Makerere College of Health Sciences, Kampala, Uganda; 3 Butabika National Psychiatric Referral Hospital, Kampala, Uganda; 4 School of Public Health, University of the Witwatersrand, Johannesburg, South Africa; 5 Senior Wellcome Trust Fellowship, London, UK

**Keywords:** Major depressive disorder, Pro-inflammatory cytokines, Acute phase proteins, HIV, TNF-α, Il-6

## Abstract

**Background:**

Major depressive disorder (MDD) is a common psychiatric complication of HIV/AIDS. While considerable research has been undertaken to understand the psychosocial risk factors of MDD, there is a paucity of data on its biological risk factors including immunological factors. To address this we undertook a study to investigate the association between MDD and pro-inflammatory cytokines and acute phase proteins among persons living with HIV/AIDS (PLWHA) in Uganda. We collected clinical and laboratory data on 201 PLWHA attending two HIV clinics in central and southwestern Uganda. Clinical data included DSM-IV based MDD diagnosis, while laboratory data included the concentrations of IL-6, TNF-α and CRP measured using ELISA. Multiple logistic linear regression analysis was used to determine which proteins were independently significantly associated with MDD controlling for study site, sex, age and highest educational attainment.

**Results:**

The prevalence of MDD was 62/201 (30.8%). Adjusting for confounders, the odds of MDD increased with increasing levels of IL-6 [each unit increase in IL-6 titres was associated with an aOR = 0.98 (95% CI, 0.97–0.99); *p* < 0.001]. Participants with low levels of TNF-α were at reduced risk of MDD compared to participants with no TNF-α [those with a TNF-α of 1- <50 pg/ml titres had an aOR = 0.35(95% CI,0.10–1.16)], but as the level of TNF-α increased, the risk of MDD increased, and in particular participants with high levels of TNF-α (of 500 or above) were at a significantly increased risk of MDD [e.g. those with a TNF-α of 500- < 1000 pg/ml titres had an aOR = 3.98 (95% CI,1.29–12.33)] compared to participants with no TNF-α. There was no evidence that MDD was associated with the level of CRP titres [aOR = 0.95 (0.78–1.15); *p* = 0.60)].

**Conclusion:**

In this study, the pro-inflammatory proteins IL-6 and TNF-α were significantly associated with MDD, while CRP was not.

## Backround

According to projections by the World Health Organization (WHO), by the year 2030 major depressive disorder (MDD) will result in more years of life lost to disability than any other illness [1]. HIV/AIDS is associated with a heavy burden of MDD with studies reporting rates of between 3 and 54% [2–8]. MDD in HIV/AIDS, apart from impairing quality of life, is associated with faster HIV diseases progression [9], poor adherence to anti-HIV medication [10], increased risky sexual behaviour [11] and may lead to mortality through its association with suicidal behaviour [2]. While a lot of work has been undertaken to understand the psychological and social risk factors of MDD in HIV/AIDS in the African context, there is paucity of data on its biological risk factors including immunological mechanisms. Understanding the immunological mechanisms underlying MDD in chronic infections such as HIV/AIDS may provide insights that may facilitate the development of new drug targets [12], biomarkers for diagnosis [13] and treatment response [14].

One of the theories that has been postulated to explain the association between MDD and immune activation following an infection like HIV is the ‘5-hydroxytryptamine’ (5-HT) hypothesis of depression [12]. According to this theory, immune activation (following an infection such as HIV/AIDS) releases pro-inflammatory cytokines such as interferon gamma (IFNγ), interleukin 6 (IL-6), and tumor necrosis factor alpha (TNF-α) which induce secretion of indoleamine 2, 3-dioxygenase (IDO). IDO activation leads to lower plasma tryptophan (a precursor of the neurotransmitter serotonin whose depletion has been implicated in MDD) and an increased synthesis of detrimental tryptophan catabolites (TRYCATs) such as kynurenine, kynurenic acid, xanthurenic acid, and quinolinic acid which have been shown to be depressogenic [12]. Immune activation including increased production of proinflammatory cytokines and acute phase proteins has repeatedly been described in MDD. Meta-analyses by both Howren and others(2009) and Dowlati and colleagues (2010) have reported an association between MDD and increased circulating peripheral concentrations of the proteins: acute phase proteins (CRP), IL-6, and interleukin 1 (IL-1) receptor antagonist and TNFα [15, 16]. We therefore hypothesised that MDD in HIV/AIDS will be associated with elevated levels of proinflammatory cytokines and acute phase proteins. To investigate this in the HIV situation of Uganda, we undertook a study among ART naïve HIV positive adults at two HIV clinics in central and south-western Uganda.

## Methods

### Study design

This was a cross-sectional study nested within a bigger study that was investigating the association between MDD and psychosocial risk factors in HIV [8, 17]. Study participants were ART naïve patients attending two specialised HIV clinics run by the AIDS Support Organisation (TASO) at Entebbe (semi-urban) and Masaka (predominantly rural) [18]. The MRC/UVRI Uganda Research Unit (the host research institution) has established research collaboration with these two study clinics. Out of 1100 participants enrolled for the main study, a random sample of 201 participants was selected using tables of random numbers to provide plasma samples for the immunological sub-study.

### Study questionnaire

A structured standardised questionnaire administered by trained psychiatric nurses was used to collect the socio-demographic and clinical data for the mother study. The following socio-demographic factors that were relevant to the immunological sub-study were collected age, gender, highest educational attainment, religion, employment status, marital status and socio-economic index (SEI) which was constructed from commonly available household items in a typical Ugandan households, with higher scores reflecting higher socio-economic status [19]. Clinical data collected included the CD4 count and an assessment for major depressive disorder (MDD) using the Mini International Neuropsychiatric interview (M.I.N.I. Plus), a structured interview based on the DSM-IV and ICD-10 classifications [20].

### Immunological assessment

Plasma IL-6, TNF-α and CRP concentrations were measured by ELISA. Anti human TNF-α (MAb TNF3/4) and IL-6 antibodies (MAb 13A5) were purchased from MABTECH AB, Sweden whereas the anti human CRP antibody (Part 842676) was purchased from Research & Diagnostics (R &D) systems, USA.

Briefly, 96-well immunolon 2HB micro plates (Thermo Labsystems, USA) were coated with anti human monoclonal antibodies (IL-6 at 0.25 μg/ml, TNF-α at 1.5 μg/ml and CRP at 2 μg/ml) at 100 μl per well and incubated over night at 4 °C. The following day, the coated plates were washed three times using 200 μl of PBS containing 0.05% Tween, pH 7.4 (Sigma-Aldrich, USA) and blocked for 1 h (at room temperature) with 200 μl of PBS and 0.05% Tween 20 (Sigma-Aldrich, USA) containing 0.1% MACS BSA buffer (Miltenyi Biotec, Germany). The stored plasma samples were thawed on the bench, diluted using the provided ELISA Diluent (1,2 for IL-6 and TNF-α)/Reagent Diluent (at 1:6400 for CRP) and 100 μl added in duplicates and then incubated at room temperature for 2 h. The plates were washed four times with 200 μl of PBS containing 0.05% Tween 20 and 100 μl of anti human IL-6 (MAb 39C3), TNF-α (MAb TNF5) or CRP (Part 842677) biotinylated detection antibodies (0.5 μg/ml for both IL-6 and TNF-α then 125 ng/ml for CRP) were added and incubated at room temperature (1 h for both TNF-α and IL-6 and 2 h for CRP). After the plates were washed as stated above, 100 μl of Streptavidin-HRP was added (diluted in incubation buffer at 1:2000 for both IL-6 and TNF-α, and 1:200 for CRP) and incubated (1 h for both IL-6 and TNF-α, and 20 min for CRP) at room temperature. After further washes as stated above, 100 μl of TMB-substrate (KPL, USA for the IL-6 and TNF-α and R & D systems, USA for CRP) was added for colour development. The assay reaction was stopped by adding 1 M sulphuric acid (100 μl to the IL-6 and TNF-α and 50 μl to the CRP) and the optical densities were measured immediately using a microplate reader (Biotek uQuant, USA) set to 450 nm. Any samples whose C.V were greater than 15% were repeated.

### Data analysis

Using STATA (version 12.0), the median concentrations of TNF-a, IL-6 and CRP were computed and compared between participants with MDD and those without MDD. Multiple logistic linear regression analysis was used to determine which inflammatory protein was independently significantly associated with MDD after controlling for study site, sex, age and highest educational attainment. A *p*-value of ≤0.05 was considered statistically significant. Additionally, using the software Prism Version 6.0 h, graphs were generated from this data.

### Ethical considerations

The study obtained ethical approval from the Uganda Virus Research Institute’s Science and Ethics Committee and the Uganda National Council of Science and Technology. Only study clinic attendees who were not impaired in anyway and had full cognitive insight were asked to participate in this study. Study participants who agreed to part in this study provided written consent after being provided with adequate information about the study. Respondents found to have significant psychiatric problems were referred to the psychiatric departments at Entebbe district hospital (at the semi-urban site) and Masaka regional referral hospital (rural site) for further assessment and management.

## Results

### Socio-demographic and clinical characteristics and their association with MDD

Table 1 shows the socio-demographic and clinical factors of the 201 participants in this study broken down by whether or not they had MDD. Overall MDD prevalence was 62 (30.8%). More than half of the participants came from TASO Masaka and participants from Masaka were more likely to have MDD than participants from TASO Entebbe (41.1% vs. 18.1%). Almost 80% of participants were female and female participants had a slightly higher prevalence of MDD than male participants (32.5% vs. 24.4%). About one third of participants were aged 18–29 years, one third were aged 30–39 and one third were aged 40 or over. There was no clear trend of MDD with age. Almost two thirds of participants had primary level as the highest level of education, while almost one quarter had secondary or higher education; those with secondary or higher education had a lower prevalence of MDD than those with primary or no education. Half of the participants were currently married; the prevalence of MDD was highest among the widowed and lowest among the currently married. Over half of the participants were Catholic; there was no clear pattern of MDD varying between different religions. Almost 37% of participants worked as traders, artisans or in the transport sector, with a further 30% involved in farming or fishing. MDD was highest among participants who were unemployed or retired. The prevalence of MDD decreased with increasing socio-economic status. About one quarter of participants had a baseline CD4 count of below 350 cells/μl (and thus qualified for ART under the then prevailing ART guidelines) with a further quarter having CD4 counts between 350 and 499 cells/μl. There was no clear pattern of MDD increasing or decreasing with CD4 count.

**Table 1:** Depression status of patients by Socio-demographic factors and CD4 counts

Factor	Level	No MDD (*n* = 139)	Has MDD (*n* = 62)	Total (*n* = 201)
Overall		139 (69.2%)	62 (30.8%)	201
Study Site	Entebbe	73 (82.0%)	16 (18.0%)	89 (44.3%)
	Masaka	66 (58.9%)	46 (41.1%)	112 (55.7%)
Sex	Male	31 (75.6%)	10 (24.4%)	41 (20.4%)
	Female	108 (67.5%)	52 (32.5%)	160 (79.6%)
Age (years)	18–29	51 (76.1%)	16 (23.9%)	67 (33.3%)
	30–34	27 (69.2%)	12 (30.8%)	39 (19.4%)
	35–39	16 (61.5%)	10 (38.5%)	26 (12.9%)
	40–49	27 (56.2%)	21 (43.8%)	48 (23.9%)
	50 +	18 (85.7%)	3 (14.3%)	21 (10.4%)
Education Level	None	15 (62.5%)	9 (37.5%)	24 (11.9%)
	Primary	86 (66.7%)	43 (33.3%)	129 (64.2%)
	Secondary or higher	38 (79.2%)	10 (20.8%)	48 (23.9%)
Marital Status	Current married	73 (72.3%)	28 (27.7%)	101 (50.2%)
	Widowed	18 (60.0%)	12 (40.0%)	30 (14.9%)
	Separated/Divorced	36 (66.7%)	18 (33.3%)	54 (26.9%)
	Single	10 (71.4%)	4 (28.6%)	14 (7.0%)
	Missing	2 (100%)	0	2 (1.0%)
Religion	Catholic	80 (66.7%)	40 (33.3%)	120 (59.7%)
	Protestant	23 (74.2%)	8 (25.8%)	31 (15.4%)
	Muslim	24 (72.7%)	9 (27.3%)	33 (16.4%)
	Seventh Day Adventist	3 (75.0%)	1 (25.0%)	4 (2.0%)
	Born Again	9 (69.2%)	4 (30.8%)	13 (6.5%)
Employment Status	Farmer/Fisherman	44 (71.0%)	18 (29.0%)	62 (30.8%)
	Professional/Clerical	9 (90.0%)	1 (10.0%)	10 (5.0%)
	Trader/Artisan/Transport	51 (68.9%)	23 (31.1%)	74 (36.8%)
	Unemployed/Retired	12 (52.2%)	11 (47.8%)	23 (11.4%)
	Student/Other	23 (71.9%)	9 (28.1%)	32 (15.9%)
Socio-economic status index	<=12	26 (57.8%)	19 (42.2%)	45 (22.4%)
	13–15	52 (68.4%)	24 (31.6%)	76 (37.8%)
	16–19	44 (73.3%)	16 (26.7%)	60 (29.8%)
	20 +	17 (85.0%)	3 (15.0%)	20 (10.0%)
CD4 count (grouped)	< 250 cells/μl	20 (76.9%)	6 (23.1%)	26 (12.9%)
	250–349	15 (60.0%)	10 (40.0%)	25 (12.4%)
	350–499	43 (76.8%)	13 (23.2%)	56 (27.9%)
	500–749	45 (68.2%)	21 (31.8%)	66 (32.8%)
	> = 750 cells/μl	16 (57.1%)	12 (42.9%)	28 (13.9%)

### Inflammatory proteins by MDD status

**Fig. 1:**
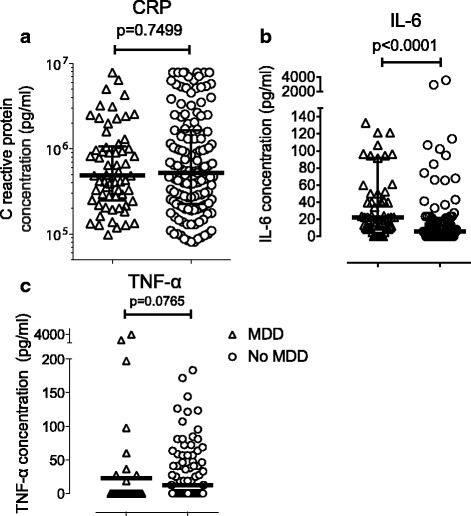
Association of CRP (**a**), IL-6 (**b**) and TNF-α (**c**) with major depressive disorder. Each symbol (triangle or circle) represents CRP, IL-6 or TNF-α concentration in the plasma of each participant. Within each data cluster for MDD or No MDD, the middle line is the median and outer lines are the interquartile range

Figure 1 shows the bivariate associations between the inflammatory proteins and MDD. The IL-6 concentration was significantly higher among MDD participants compared to those without MDD (*p* < 0.0001; Median 22.4 pg/ml for MDD participants *n* = 62 and 5.762 pg/ml for participants with no MDD *n* = 139; Fig. 1B). There was a trend suggesting higher TNF-α concentrations among MDD participants compared to those without MDD (*p* = 0.0765; median 22.98 pg/ml for MDD participants n = 62 and 12.41 pg/ml for participants with no MDD *n* = 139; Fig. 1C). There was no trend between CRP and MDD (*p* = 0.7499; Fig. IA).

### Multivariate analysis of the association between immunological proteins and MDD

According to Table 2, participants in Masaka were more likely to have MDD than those in Entebbe. There was no evidence that gender, age or education level influenced MDD. Adjusting for the confounders there was no evidence that MDD was associated with the level of CRP. IL-6 and TNF-α were associated with MDD (Table 2) in that the odds of MDD increased with increasing IL-6, but in a non-linear fashion. Having any TNF-α decreases the odds of MDD, but as the level of TNF-α increases, the odds of MDD increase. Participants with low levels of TNF-α showed reduced risk of MDD compared to participants with no TNF-α, but as the level of TNF-α increase, the risk of MDD increases, and in particular participants with high levels of TNF-α (of 500 or above) were at a significantly increased risk of MDD. In summary, the odds of MDD increased with increasing levels of IL-6; participants with low levels of TNF-α were at reduced risk of MDD compared to participants with no TNF-α, but as the level of TNF-α increased, the risk of MDD increased, and in particular participants with high levels of TNF-α (of 500 or above) were at a significantly increased risk of MDD.

**Table 2:** Multiple logistic linear regression model for Major depressive disorder

Factor	Level	aOR (95% CI)	LR *P*-value
Study Site	Entebbe	1 (Reference Level)	0.003
	Masaka	3.30 (1.46; 7.45)	
Sex	Male	1 (Reference Level)	0.48
	Female	1.40 (0.54; 3.66)	
Age (years)	Per one year increase	0.981 (0.944; 1.020)	0.32
Education Level	None	1 (Reference Level)	0.88
	Primary	1.16 (0.38; 3.51)	
	Secondary	0.93 (0.25; 3.41)	
CRP	Per 1 million increase	0.95 (0.78; 1.15)	0.60
100/(IL-6 + 1)	Per unit increase	0.979 (0.969; 0.989)	<0.001
TNF-α	0	1 (Reference Level)	<0.001
	1 - <50	0.35 (0.10; 1.16)	
	50 - <500	0.31 (0.10; 0.94)	
	500 - <1000	3.98 (1.29; 12.33)	
	1000 +	7.24 (1.65; 31.82)	

## Discussion

This is one of the first studies from sub-Saharan Africa that has investigated the association between pro-inflammatory cytokines and acute phase proteins and major depressive disorder (MDD) in HIV. In this study, IL-6 was significantly independently positively associated with MDD. An increase in the risk of MDD with increasing levels of IL-6 mRNA has been reported in previous studies undertaken in the developed world both among persons living with HIV (PLWHA) [21] and in other medical conditions such as cancer [22], cardiovascular disease, osteoporosis [23] and among the elderly [24]. In this study, TNF-α was significantly independently positively associated with MDD. In a systematic review that involved 13 studies, Dowlati and colleagues (2010) reported a statistically significant positive association between TNF-α levels and MDD [16]. In this study, while the overall positive association between TNF-α levels and MDD was true at TNF-α concentrations above 500 pg/ml, the relationship between TNF-α and MDD was the reverse at TNF-α concentrations below 500 pg/ml. This non-linear association between TNF-α and MDD has not previously been reported.

In this study, there was no association between C-reactive protein (CRP) and MDD. Previous studies have reported conflicting results on the association between CRP and MDD, while some studies have observed a positive association between CRP and depressive symptoms [15, 25–27], others have not [28]. The relationship between CRP and MDD appears to be further complicated by sex and race. Morris and colleagues (2011) in the USA observed a positive association between CRP and MDD among female Caucasians but not among male Caucasians and not among African Americans of either sex [26]. Therefore the lack of association between CRP and MDD observed in this study could be due to race differences. There is however need for more African studies to confirm this finding.

## Conclusion

In conclusion, as hypothesized in this study, MDD was generally associated with elevated pro-inflammatory proteins. These results suggest a possible aetiological role for pro-inflammatory proteins in MDD. But since this study was cross-sectional in design, we could not determine the direction of causality between the pro-inflammatory proteins and MDD. Secondly, since we assumed that HIV and MDD are each independently associated with inflammation, an HIV negative control group would have been required in order to investigate the independent effect of MDD on inflammation. To resolve these issues will in future require the use of a longitudinal study design in order to delineate the actual direction of causality and the inclusion of an HIV negative control group. However contrary to the set hypothesis, MDD was not associated with acute phase proteins. As discussed above this may have been due to differences in race.

## Abbreviations

AIDS: Acquired immunodeficiency syndrome
ART: Antiretroviral therapy
CRP: C-reactive protein
DSM-IV: Diagnostic and Statistical Manual for Mental Disorders, 4th edition
EDCTP: European and developing countries’ clinical trials partnership
ELISA: Enzyme linked immunosorbent assay
HIV: Human immunodeficiency virus
ICD: International Classification of Diseases
IDO: Indoleamine 2,3-dioxygenase
IgG: Immunoglobulin Gamma
IL: Interleukin
M.I.N.I: Mini International Neuropsychiatric interview
mAb: Monoclonal antibody
MDD: Major depressive disorder
mRNA: Messenger ribonucleic acid
PLWHA: Persons living with HIV/AIDS
TASO: The AIDS Support Organization
TNF-α: Tumour necrosis factor alpha
TRYCATs: Tryptophan catabolites
WHO: World Health Organization
